# Evaluating Vitamin D and *foxp3* mRNA levels in women with recurrent spontaneous abortion

**DOI:** 10.5935/1518-0557.20210062

**Published:** 2022

**Authors:** Fatemeh Dehghani Tafti, Fateme Zare, Seyed Mohsen Miresmaeili, Farzaneh Fesahat

**Affiliations:** 1 Department of Biology, Science and Arts University, Yazd, Iran; 2 Reproductive Immunology Research Center, Shahid Sadoughi University of Medical Sciences, Yazd, Iran

**Keywords:** recurrent spontaneous abortion, 1,25-dihydroxyvitamin D3, Forkhead 3 box protein

## Abstract

**Objective:**

This current survey investigated the role of the Forkhead 3 box protein (foxp3) gene and serum vitamin D levels in women with recurrent spontaneous abortion (RSA).

**Methods:**

The mRNA level of the *foxp3* gene in peripheral blood was evaluated in women with a history of RSA (N=40) and in controls (N=40) via quantitative polymerase chain reaction. We employed the enzyme-linked immunosorbent assay to assess the serum levels of 1,25-dihydroxyvitamin D3 (1,25(OH)2 D) in both groups. The Mann-Whitney U test and Pearson's correlation coefficient were used to statistically compare study groups between and within themselves, respectively.

**Results:**

Although mRNA levels of foxp3 were higher in women with RSA than in controls, we observed no significant change in mRNA levels of foxp3 between the two groups (*p*=0.16). An important positive correlation was observed between foxp3 mRNA levels and 1,25(OH)2 D in controls (*p*=0.003). In contrast, the correlation between foxp3 expression and 1,25(OH)2 D was not significant in the case group (*p*=0.14). Serum vitamin D levels were lower in women with RSA than in controls (*p*<0.001).

**Conclusions:**

Our ﬁndings demonstrated that 1,25Vitamin D3 along with other molecules might help prevent RSA by providing for an anti-inflammatory state not necessarily through foxp3 expression or T cell differentiation.

## INTRODUCTION

Recurrent spontaneous abortion (RSA) refers to the recurrent loss of a fetus (bodyweight <1000 g) occurring before the 20^th^ week of pregnancy in more than two occasions with the same sexual partner (Practice Committee of the American Society for Reproductive Medicine, 2020). About 1-5% of all women of reproductive age suffer from RSA (Kim *et al*., 2004; Pildner von Steinburg & Schneider, 2009). Early pregnancy failure is a prevalent issue accounting for 15% to 20% of known clinical pregnancies and miscarriages. (Chen & Creinin, 2007). Numerous causes for RSA have been described, including anatomical, immune, and genetic factors (Practice Committee of the American Society for Reproductive Medicine, 2012; Motedayyen *et al*., 2018; Talebi *et al*., 2016). The cause of RSA remains unexplained in some cases (Ford & Schust, 2009; Laird *et al*., 2003).

Vitamin D is a secosteroid hormone with a fundamental role in bone metabolism and mineral homeostasis as well as immune system modulation (Tamblyn *et al*., 2015; Lagishetty *et al.,* 2011). Evidence indicates that Vitamin D may be involved in the pathogenesis of RSA (Triggianese *et al*., 2016). Its deficiency has a remarkable impact on pregnancy outcomes and has been linked to RSA, low birth weight, poor growth, fragile ossification, and increased risk of autoimmune disorders (Tabesh *et al*., 2013). Indeed, several pregnancy complications including gestational diabetes, preeclampsia, maternal infection, cesarean section, and preterm labor have been considered to result from vitamin deficiency (Tabesh *et al*., 2013; Theodoratou *et al*., 2014). The extra 1- alpha-hydroxylase activity released from the decidua, placenta, and both fetal and maternal kidneys increase the metabolism of vitamin D during pregnancy (Sanz-Salvador *et al*., 2015). Vitamin D affects bone metabolism more than its classic counterparts (Christakos *et al*., 2016; Baeke *et al*., 2010). There is a growing trend of reusing vitamin D to reduce the regulation of pathological immune responses in patients with autoimmune or inflammatory diseases. Higher levels of vitamin D may trigger different anti-inflammatory functions, which contain the function of T regulatory cells (Tregs) and/or increase their numbers. Other small molecules such as niacin, short-chain fatty acids, and vitamin A may gain Tregs (Sakaki *et al*., 2005; Wacker & Holick, 2013). High levels of *1*,25-dihydroxyvitamin *D3 (1*,25(OH)2 *D)* may affect the lineage-specific foxp3 transcription factor involved in the function and creation of Tregs (Joshi *et al*., 2011; Jeffery *et al*., 2009). High levels of 1,25(OH)2D have been related to anti-inflammatory lymphoid polarization containing large amounts of Tregs (Dimeloe *et al*., 2010).

Forkhead 3 box protein (foxp3) is a nuclear transcription factor required to induce immunosuppressive activity (Hu *et al*., 2019). The role and function of foxp3 in tumorigenesis is conflicting. Expression of *foxp3* in tumor cells plays an important role in tumorigenesis (Zuo *et al.*, 2007a; b). Selective expression of foxp3 in human trophoblasts may be related to the multiplication and aggressive behavior of trophoblasts (Dimeloe *et al*., 2010). Foxp3 is known as the marker of regulatory T cells (Treg), CD4 + CD25 + and is a major determinant of immune function. Due to the strong immunosuppressive effect of foxp3 in Treg cells, *foxp3* in trophoblasts may have immunosuppressive impacts analogous to those seen in Treg cells, and the function of Treg cells may be a new mechanism of maternal and embryonic endurance (Hu *et al*., 2019; Niu *et al*., 2011). As shown by the role of *foxp3* expression in tumor cells and tumorigenesis, foxp3 may play an analogous role in trophoblasts.

However, to our knowledge, there are no reports on serum vitamin D levels and *foxp3* expression patterns in women with a history of RSA. Therefore, the main goal of this prospective study was to investigate the serum concentration of vitamin D and *foxp3* mRNA levels in women with a history of recurrent miscarriage compared to healthy fertile women with at least one successful pregnancy and live birth.

## MATERIAL AND METHODS

### Study population and sample collection

This case-control study was based on blood samples taken from women referred to the Yazd Institute of Reproductive Sciences, Iran. The Ethics Review Board of the Science and Arts University of Yazd approved this study. Informed consent was obtained from all participants included in the study. The procedures involving humans performed during the course of the study met the ethical standards of our Institution and/or the National Research Committee and the requirements set out in the 1964 Helsinki Declaration and later amendments made thereto.

The control group consisted of 40 pregnant women without a history of miscarriage and at least one successful pregnancy. The case group included 40 women with a history of two or more cases of RSA (Maesawa *et al*., 2015). The groups were matched for age. A gynecologist monitored the women in the case group. No male factor etiologies, anatomical or endocrinal complications were allowed in the study; patients had normal parental chromosomal karyotypes, lack of anti-phospholipid antibodies (IgG and IgM classes), no thrombophilia, TORCH syndrome negativity, anti-cardiolipin antibodies (IgG, IgA, and IgM classes) were considered as inclusion criteria of two groups. Samples of 5 ml of peripheral blood were taken from each study participant; half of the samples were used in gene expression evaluation and half in vitamin D level testing.

### Enzyme-linked immunosorbent assay

The 2.5-ml blood samples of each participant were separated after clotting and serum was removed. Enzyme-linked immunosorbent assay (ELISA) test kits were used to specify the serum concentrations of *1*,25(OH)2 *D* in the two groups according to the manufacturer's instructions (Monokit, IRAN). The detection limit of *1*,25(OH)2 *D* or each sample was 1.2 ng/ml.

### RNA extraction and cDNA synthesis

Two ml of intravenous blood samples taken from the study population were transferred to tubes containing ethylenediaminetetraacetic acid. Then, the total RNA of each sample was extracted using the easy cDNA Synthesis kit (Parstous biotechnology, Iran). The purity and concentration of extracted RNAs were determined using a spectrophotometer in the absorbance of the A260 / A280 ratio and 260 nm, respectively (PhotoBiometer, Eppendorf, Germany). All complementary DNA (cDNA) was synthesized from 1µg RNA with the Revert Aid First Strand cDNA Synthesis Kit (parstous biotechnology, Iran). The cDNA product was kept at -20°C until use.

### Gene expression assessment

Quantitative real-time polymerase chain reaction (qRT-PCR) was used to determine the mRNA levels of *foxp3* in case and control groups. *GAPDH* was considered as a reference gene for the normalization of *foxp3* expression levels. The primer sequences used in this study are presented in Table1. Master Mix Green with high ROX(tm) (Amplicon) was utilized for PCR reaction in a StepOne system (Applied Biosystems, CA, USA). Each PCR run was performed in a ﬁnal volume of 20 µL containing, cDNA (2 µL), forward primer (1 µL), reverse primer (1 µL), the master mix (10 µL), and 6 µL nuclease-free water. All run methods consisted of one cycle of holding stage (10 min at 95°C), followed by 40 cycles in the amplification stage at 95°C for 15 s, 60°C for 30 s, and 72°C for 30 s. A melting curve stage was run after the cycling stage in the range of 60°C to 95°C to verify amplicon speciﬁcity. The relative expression level of each gene was analyzed using the 2-∆∆Ct method.

**Table 1.** Primer sequences used for the real-time polymerase chain reaction.

Gene	sequence (5’-3’)	Accession No.	PCR product (bp)
***Foxp3***	F: ACC TGG AAG AAC GCC ATC R: TGT TCG TCC ATC CTC CTT TC	NM_014009.4	192
***GAPDH***	F:AAA TCA AGT GGG GCG ATG CTG R: GCA GAG ATG ATG ACC CTT TTG	NM_001357943.2	118

PCR, polymerase chain reaction; F, forward; R, reverse; *Foxp3, forkhead box P3; GAPDH, glyceraldehyde-3-phosphate dehydrogenase*

### Statistical Analysis

SPSS 20 (Chicago, IL, USA) was used in statistical analysis. The Mann-Whitney U test was applied to compare the variables between the case and control groups. Pearson's correlation coefficient was used to measure the correlation between gene expression and vitamin D levels in each group. The data was presented as mean ± standard deviation and *p*<0.05 was deemed significant.

## RESULTS

**Figure 1:**
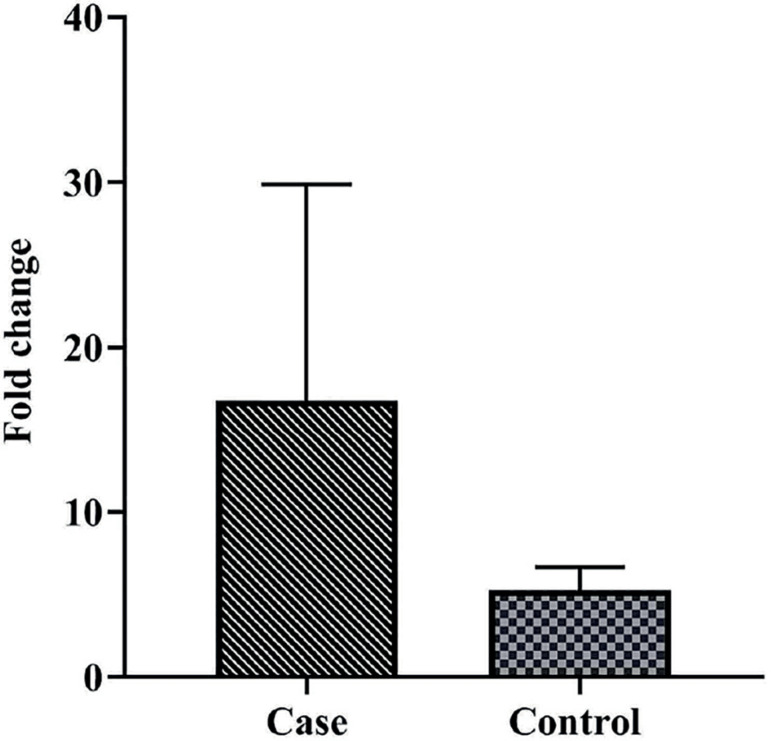
Comparison of *foxp3* gene expression in cells obtained from blood samples of women with a history of recurrent spontaneous abortion (case) and healthy fertile women (controls) There was no significant fold change in *foxp3* mRNA levels between case and control groups (*p* value=0.16, Mean ± SEM: 16.87±13.11 *versus* 5.26±1.39, respectively).

**Figure 2:**
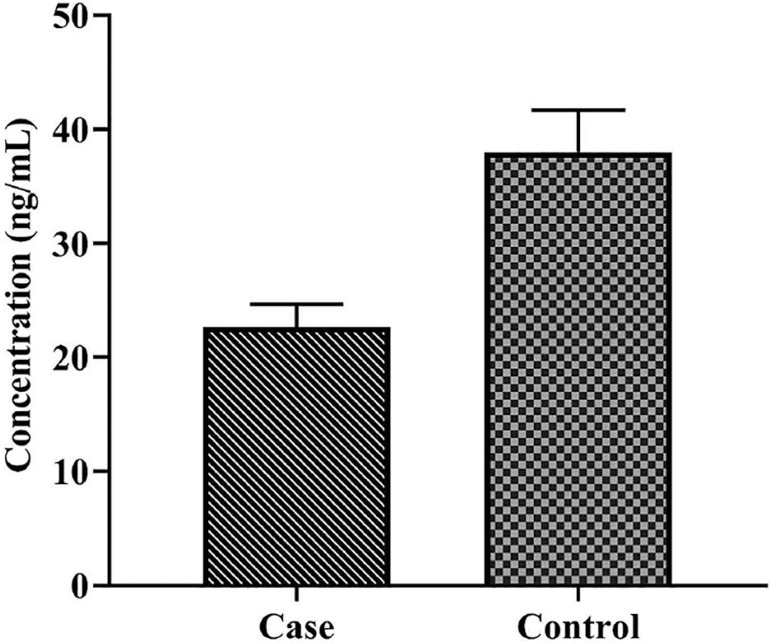
Comparison of vitamin D in serum from blood samples of women with a history of recurrent spontaneous abortion (case) and healthy fertile women (controls) There was a significantly lower level of vitamin D in the serum samples of case group subjects compared to controls (*p* value ≤0.001, Mean ± SEM: 21.39±1.94 versus 36.84±3.97, respectively).

A total of 40 cases and 40 healthy controls were included in this study. The demographic features of the two study groups are summarized in Table 2. As mentioned before, women in the case group were matched in regards to age with any significant differences than those in the controls (*p*≥0.05). The mRNA levels of *foxp3* were higher in women with a history of RSA than in controls; however, there was no significant fold change in *foxp3* mRNA levels between patients in the case and control groups (*p*=0.16, Mean ± SEM: 16.87±13.11 *vs*. 5.26±1.39, respectively) (Figure 1). There was a significant direct correlation between *foxp3* expression and *1*,25(OH)2 *D* in controls (*p*=0.003, R=0.49). In contrast, the correlation between *foxp3* gene expression and *1*,25(OH)2 *D* levels was not significant in the case group (*p*=0.14, R=-0.25) (Table 3). *1*,25(OH)2 *D* levels were significantly lower in the serum samples of women with a history of RSA compared to controls (*p*<0.001, Mean ± SEM: 21.39±1.94 *versus* 36.84±3.97, respectively) (Figure 2).

**Table 2.** Demographic data of the study groups.

Groups	Age (Year)	Number of miscarriages	Successful pregnancies
**Case (N=40)**	31±3.78	4.0±0.9	0
**Control (N=40)**	31.06±3.43	0	2.12±0.93

Data are presented as mean ±S.D.

Case; Recurrent spontaneous abortion, Control; Healthy fertile women

**Table 3.** Correlation between Vitamin D and FOXP3 expression in case and control groups.

Groups	Variable	*FOXP3* expression
**Case**	**Vitamin D**	R=-0.25 P=0.14 CI=-.0.53--0.08
**Control**		R=0.49 **P=0.003** CI= 0.18-0.7

*Foxp3, forkhead box P3*. Pearson’s correlation coefficient was used.

## DISCUSSION

The cause of RSA, a negative pregnancy outcome that occurs in women around the world, is related to endocrine abnormalities, thrombophilic disorders, genetic factors, and anatomical abnormalities. However, the causes of most cases of RSA are unknown and may relate to immune factors (Wolf *et al*., 2005).

Poor vitamin D levels have been associated with pregnancy loss, recurrent implantation failure, and pregnancy-related disorders such as preeclampsia (Schröder-Heurich *et al.*, 2020). This study found significantly lower concentrations of vitamin D in the blood serum samples of patients with a history of RSA compared to controls. Li *et al*. (2017) assessed the concentration of vitamin D and the expression of vitamin D receptors in the decidual tissues of women with a history of RSA (N=30) and women in early pregnancy undergoing elective abortion as controls (N=30). As seen in our study, individuals with a history of RSA had significantly lower levels of vitamin D and its receptor in deciduous tissues compared to controls, indicating that vitamin D has a role in the production of inflammatory cytokines and possibly in the etiology of RSA (Li *et al*., 2017). Sharif *et al*. (2018) assessed the relationship between vitamin D, recurrent pregnancy loss, and autoimmunity. According to the authors, during pregnancy, fetal endurance can occur through a complex interaction of various regulatory factors in the innate and adaptive immune system, which can result in miscarriage. While deficiency has been associated with pregnancy failure, vitamin D may modulate the immune response of the fetal interface and help create a more conducive environment for a successful pregnancy. The authors suggested a significant role for adequate vitamin D supplementation in preventing recurrent miscarriage (Sharif *et al*., 2018). Two reviews conducted by Gonçalves *et al*. (2018) and Zhang *et al*. (2017) concluded that vitamin D might be essential in the immune system, promoting implantation and successful pregnancy. Also, women with a history of RSA with low levels of vitamin D appear to have more autoimmune and cellular immune abnormalities.

Lack of Vitamin D suggests that pre-pregnancy vitamin D supplementation in women may affect glucose-induced tumor necrosis factor receptors such as *foxp3* and glucocorticoid receptors protect against RSA (Abdollahi *et al*., 2020a). One research showed that *foxp3* was also expressed in human trophoblasts in addition to Treg cells and abnormal expression of *foxp3* in trophoblasts may relate to RPL (Hu *et al*., 2019). Hu *et al*. (2019) used RT-qPCR to study the *foxp3* expression profile in the trophoblasts of women with recurrent pregnancy loss. They concluded that *foxp3* is expressed in trophoblasts and is reduced in recurrent pregnancies. Thus, regulation of *foxp3* may help maintain maternal tolerance and fetal growth (Hu *et al*., 2019). Selective expression of *foxp3* in human trophoblasts suggests that *foxp3* expression may be associated with aggressive behavior of trophoblasts and proliferation. Abdollahi *et al.* (2020b) reported an increase in *foxp3* gene expression in the peripheral blood mononuclear cells (PBMCs) of women with unexplained recurrent pregnancy loss (URPL) in the presence of vitamin D3 compared to untreated PBMCs. In contrast, there was no significant change in the control group regarding *foxp3* gene expression in the PBMCs in the presence of 1,25Vitamin D3 compared to untreated PBMCs. They also showed that *foxp3* gene expression decreased significantly in PBMCs in women with URPL compared to controls (Abdollahi *et al*., 2020b). Unlike the abovementioned studies, in this study, the level of *foxp3* mRNA was increased in the blood of women with a history of RSA compared to controls, albeit not significantly. Also, we detected no significant direct correlation between 1,25 Vitamin D3 concentrations and *foxp3* expression in the case group. Despite the enhanced *foxp3* expression in CD4+ T cells by 1, 25(OH) 2D and the capacity of this vitamin to regulate gene expression via direct binding to the genes, it is unknown whether 1,25(OH)2D3 may directly induce *foxp3* gene expression without involving other molecules (Kang *et al*., 2012). Based on the findings of this study, 1,25 (OH) 2VD3 may, along with other molecules, alter *foxp3* expression (Kang *et al*., 2012).

In *in-vitro* cultures of human CD4 + CD25-T cells and plain putative T cells, 1, 25(OH) 2D3 was reported to increase the frequency of activation-induced foxp3 + T cells, depending on the presence of IL-2 (20). However, a comparative study using naive mouse T cells showed that 1,25- (OH) 2D3 inhibited both IL-17 and Treg differentiation in vitro (Chang *et al*., 2010).

In conclusion, our ﬁndings demonstrated that 1,25Vitamin D3 along with other molecules might act to prevent RSA by providing an anti-inflammatory state not necessarily through *foxp3* expression and T cell differentiation.
